# Uremia increases QRS duration after *β*‐adrenergic stimulation in mice

**DOI:** 10.14814/phy2.13720

**Published:** 2018-07-08

**Authors:** Morten B. Thomsen, Morten S. Nielsen, Annemarie Aarup, Line S. Bisgaard, Tanja X. Pedersen

**Affiliations:** ^1^ Department of Biomedical Sciences Faculty of Health and Medical Sciences University of Copenhagen Copenhagen Denmark

**Keywords:** Conduction velocity, electrocardiogram, renal disease, sympathetic

## Abstract

Chronic kidney disease (CKD) and uremia increase the risk of heart disease and sudden cardiac death. Coronary artery disease can only partly account for this. The remaining mechanistic links between CKD and sudden death are elusive, but may involve cardiac arrhythmias. For the present study, we hypothesized that a thorough electrophysiological study in mice with CKD would provide us valuable information that could aid in the identification of additional underlying causes of sudden cardiac death in patients with kidney disease. Partial (5/6) nephrectomy (NX) in mice induced mild CKD: plasma urea in NX was 24 ± 1 mmol/L (*n* = 23) versus 12 ± 1 mmol/L (*n* = 22) in sham‐operated control mice (*P* < 0.05). Echocardiography did not identify structural or mechanical remodeling in NX mice. Baseline ECG parameters were comparable in conscious NX and control mice; however, the normal 24‐h diurnal rhythm in QRS duration was lost in NX mice. Moreover, *β*‐adrenergic stimulation (isoprenaline, 200 *μ*g/kg intraperitoneally) prolonged QRS duration in conscious NX mice (from 12 ± 1 to 15 ± 2 msec, *P* < 0.05), but not in sham‐operated controls (from 13 ± 1 to 13 ± 2 msec, *P *>* *0.05). No spontaneous arrhythmias were observed in conscious NX mice, and intracardiac pacing in anesthetized mice showed a comparable arrhythmia vulnerability in NX and sham‐operated mice. Isoprenaline (2 mg/kg intraperitoneally) changed the duration of the QRS complex from 11.2 ± 0.4 to 11.9 ± 0.5 (*P *= 0.06) in NX mice and from 10.7 ± 0.6 to 10.6 ± 0.6 (*P *= 0.50) in sham‐operated mice. Ex vivo measurements of cardiac ventricular conduction velocity were comparable in NX and sham mice. Transcriptional activity of Scn5a, Gja1 and several profibrotic genes was similar in NX and sham mice. We conclude that proper kidney function is necessary to maintain diurnal variation in QRS duration and that sympathetic regulation of the QRS duration is altered in kidney disease.

## Introduction

More than 20 million Americans are living with chronic kidney diseases (CKD) and eight million of these have moderate or severe CKD (Levey et al. 2003). Having CKD places the patient at increased risk of sudden cardiac death (Sarnak et al. 2003; Weiner et al. 2004; Pun et al. 2009; Green and Roberts 2010; Herzog et al. 2011). Twenty‐seven percent of the all‐cause mortality and two‐thirds of all cardiac death in dialysis patients are due to cardiac arrhythmia and arrest (Poulikakos et al. 2014).

Coronary atherosclerosis is often found in patients with CKD and is associated with a higher frequency of arrhythmic death than in the general population (Herzog et al. 2011). Nevertheless, dialysis patients undergoing cardiac revascularization after myocardial infarction do not have reduced probability of sudden cardiac death compared to the population of dialysis patients as a whole (Herzog et al. 2008). The conclusion from this study was that despite coronary intervention, a large cardiac mortality in dialysis patients remains present and the mechanism for this is unknown. In support, statin treatment to reduce atherosclerotic events in patients with CKD does not reduce risk of cardiac arrhythmic death (Baigent et al. 2011). Thus, the increased risk of cardiac arrhythmia in dialysis patients appears to be independent of coronary artery disease.

The mechanistic link between CKD and cardiac arrhythmias is multifactorial, including not only atherosclerotic disease, but also autonomic and electrolyte imbalances, hypertension, inflammation, and cardiac remodeling (Green and Roberts 2010). Establishing potential links between CKD and sudden cardiac death in animal models has clear advantages, since such studies may guide subsequent discovery of potential new treatment modalities and design of clinical trials. Rats with polycystic kidney disease due to an inherited samcystin protein defect have severely reduced glomerular filtration rate at 35 weeks of age and elevated blood urea levels (Hsueh et al. 2014). Isolated hearts from these rats show cardiac hypertrophy and delayed repolarization. Importantly, the hearts isolated from rats with kidney disease were more vulnerable to pacing‐induced arrhythmias (Hsueh et al. 2014). Contrary, Donohoe and colleagues found increased repolarizing potassium currents and faster repolarization in disaggregated cardiomyocytes from rats with a surgically induced renal failure (5/6 nephrectomy) (Donohoe et al. 2000a,b). Thus, the findings on cardiac repolarization from the electrophysiological examination in rodent models of chronic renal disease are conflicting.

Mouse models of subtotal nephrectomy offer the advantage of testing disease progression or severity. For example, Bro et al. (2008) showed that 5/6 nephrectomy does not change ventricular contractile function in apolipoprotein‐E‐deficient mice. In the present study, we aimed to document the cardiac electrophysiological effects of subtotal nephrectomy in wild‐type mice. We induced chronic kidney disease by a two‐stage 5/6 nephrectomy and compared these to sham‐operated control littermates. We recorded and analyzed 24‐h electrocardiograms from conscious mice and performed cardiac electrophysiological investigations in anesthetized mice and in isolated heart tissue.

## Methods

### General

The experiments performed in this study were approved by the national ethics committee (Ministry of Environment and Food, Denmark, permission no. 2013‐15‐2934‐00773), and conform to the European Parliament Directive on the Protection of Animals Used for Scientific Purposes (2010/63/EU). The experiments were carried out in accordance with the guidelines made by the institutional and national animal care and use committees. Male C57BL/6j Bom mice (Taconic, Ejby, Denmark) were used in all experiments. They were housed in groups in standard cages in an air‐conditioned room (21–23°C) with a 12‐h light/dark schedule, and had access to standard chow and water ad libitum.

### Renal surgery to induce chronic kidney disease

**Figure 1:**
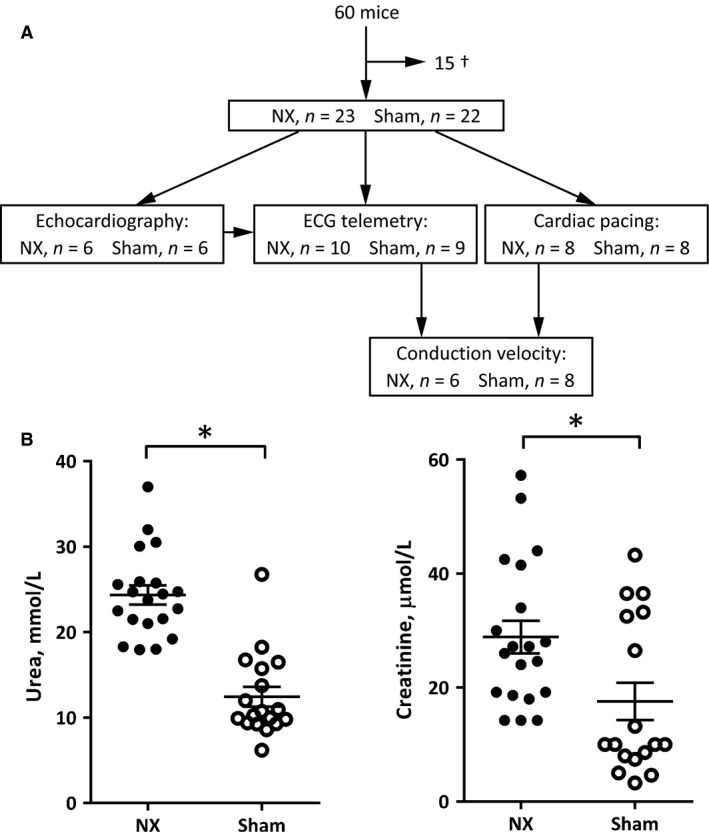
(A) Male mice were subjected to 5/6 nephrectomy (NX) or sham operation. A period of 8–9 weeks later, echocardiography, ECG telemetry, cardiac pacing, and conduction‐velocity measurements were performed on the indicated number of mice. Some, but not all mice were used for more than one test. (B) Plasma markers of kidney function, urea, and creatinine, were measured in plasma samples taken from NX (black circles) and sham‐operated (white circles) mice at the termination of the study. *P < 0.05 (Student's *t* test).

Uremia was induced in mice by 5/6 nephrectomy (NX) in a two‐step surgical procedure at 9–11 weeks of age (Fig. 1A). Surgery was performed under general anesthesia (in *μ*g/g body weight administered subcutaneously (s.c.): tiletamin 16.3; zolazepam 16.3; xylazin 26.1; butorphanol tartrate 0.65). After induction of general anesthesia, a dorsal midline incision was made and the left kidney was exposed (sham) or resected after ligation of the renal vessels and the ureter (NX) through a dorso‐ventral incision in the muscles and fascia. The muscle‐fascial incision was closed using 4‐0 suture, and the skin incision using 3‐0 suture. One week later, the upper and lower poles of the right kidney were exposed (sham) or resected (NX) on all mice in a similar procedure. Analgesia (buprenorphine 0.1 *μ*g/g body weight) was administered s.c. twice daily for up to 3 days after the surgical procedures. Mice were numbered by ear clippings, and investigators were blinded to whether the mice were nephrectomized or sham‐operated.

### Echocardiography

Transthoracic echocardiography was performed 8–9 weeks after renal surgery with a linear 30‐MHz transducer (VisualSonics, Toronto, Canada) at an axial resolution of 55 *μ*m and a lateral resolution of 115 *μ*m. Each mouse was anesthetized with 2% isoflurane in a mix of O_2_ and N_2_ (30:70) and placed in a supine position on a heated table. Posterior and anterior wall thickness and left ventricular inner diameter (LVID) were determined at systole (s) and diastole (d) from M‐mode parasternal short‐axis and long‐axis views. Left ventricular end‐diastolic and end‐systolic volumes (EDV and ESV, respectively) were estimated from LVID according to the algorithm of the manufacturer. Stroke volume (SV* *= EDV − ESV), ejection fraction (EF* *= SV/EDV), and fractional shortening (FS* *= (LVIDd − LVIDs)/LVIDd) were calculated.

### Radiotelemetry recording of ECG and β‐adrenergic challenge

Radiotransmitter telemetry devices (ETA‐F10, Data Sciences International, St. Paul, MN) were implanted in anesthetized mice (2% isoflurane in 100% O_2_) subcutaneously on the dorsal area between the scapulae, as previously described (Gottlieb et al. 2017). Postoperative analgesia included carprofen (10 mg/kg, s.c.), lidocaine, and bupivacain (0.4 and 1.0 mg/kg, respectively, in the s.c. pocket). Electrocardiograms (ECG) were recorded continuously at 1 kHz for 24 h in freely moving and conscious mice >7 days after surgery using a setup previously described (Gottlieb et al. 2017). The mice were in their normal 12‐h light/dark schedule, and had access to food and water ad libitum during the recordings. After 24‐h, a single dose of isoprenaline (200 *μ*g/kg) was administered intraperitoneally and the electrophysiological effects were recorded for 30 min. ECGs were analyzed every hour and the diurnal rhythms of the electrophysiological parameters were described by a cosinor fit to the data. Heart‐rate variability was quantified according to the recommendations by Thireau and colleagues (Thireau et al. 2008).

### Invasive electrophysiological study

Mice were anesthetized using 2% isoflurane in a mix of O_2_ and N_2_ (30:70) and a standard 6‐lead surface ECG was recorded for 10 min before any surgery (LabChart, ADInstruments, Australia). Body temperature was monitored and kept at 36–38°C during the entire procedure. The right jugular vein was isolated via a small opening of the skin on the frontal neck region, and a 1.1 F octal polar pacing catheter was inserted and advanced to the right ventricle via the right atrium. Pacing stimuli (1 msec duration) were provided by an external stimulator (DS3, Digitimer, Welwyn Garden City, UK) controlled by LabChart software and delivered at the endocardium of the right ventricle. ECGs from anesthetized and conscious mice were analyzed as described previously (Speerschneider and Thomsen 2013; Speerschneider et al. 2013).

Excitation threshold was determined by gradually increasing current amplitude until cardiac capture. Subsequent pacing was performed at four times capture threshold. After a 5‐min recording period in spontaneous sinus rhythm, we paced the right ventricle for 2 min at a constant cycle length of 80 msec. Effective refractory period of the ventricles was determined using an S1–S2 protocol, where 10 S1 stimuli with 100‐msec interpulse interval was followed by a single S2 stimulus where the S1–S2 interval was increased from 20 to 80 msec in steps of 1 msec. Arrhythmia vulnerability was assessed using a modified S1–S2 protocol with six times S2 stimuli. Burst pacing at 50 Hz for 1 sec was also used to test arrhythmia risk. After one round of pacing, isoprenaline (2 mg/kg) was administered intraperitoneally and the pacing was repeated. At the end of the experiments, a blood sample was taken and the heart was explanted for ex vivo experiments. The mice used in the invasive study were different from the mice used in the experiments employing radiotelemetry recordings of the ECG in conscious mice.

### Biochemical measurements

Blood was collected in heparinized microtubes and centrifuged at 1500 *g* for 10 min at 4°C. Plasma concentrations of urea, creatinine, phosphate, and calcium were measured on a Cobas 8000 modular analyzer series (Roche, Denmark).

### Ex vivo measurement of conduction velocity

Conduction velocity was measured essentially as described for rat atrial tissue previously (Olsen et al. 2011). In short, a longitudinal strip of the right ventricular free wall was isolated. A loop was sutured in each end of the strip and mounted in a 1‐mL chamber, perfused at 2 mL/min with a solution containing (in mmol/L): NaCl 136, KCl 4, MgCl_2_ 0.8, CaCl_2_ 1.8, HEPES 5, MES 5, Glucose 10, pH 7.4 and equilibrated with 100% O_2_. The remainder of the heart was snap frozen in liquid N_2_ and stored at −80°C until further analysis.

Strips were stimulated at the apical end with a unipolar electrode at 5 Hz (duration 0.5 msec and double threshold) and local activation was detected at two points using platinum/iridium electrodes (PI20030.5B10, Micro Probe Inc., Gaithersburg, USA) connected to two Iso‐DAM8A amplifiers (World Precision Instruments, Sarasota, USA). Signals were band‐pass filtered (0.3–10 kHz) and sampled at 30 kHz (Digidata 1322A, Axon Instruments, Union City, USA). Interelectrode distance was measured using a microscope with an ocular grid (Wild M38, Heerburg, Switzerland). Time of local activation was determined by a custom written script in MATLAB and conduction velocity was calculated as electrode distance divided by interelectrode delay.

### Gene‐expression studies

The heart was quickly thawed in 0.9% saline and a 1‐mm wide transmural strip from the left ventricular free wall was excised. RNA was extracted using the Trizol reagent as previously described (Pedersen et al. 2013). cDNA was constructed from 500 ng RNA using the High Capacity cDNA Reverse Transcription kit according to the manufacturer's instructions (Applied Biosystems). Two ng cDNA was used for quantitative RT‐PCR analyses.

Standard real‐time PCR using the fast SYBR green master mix (Applied Biosystems) was used to quantify expression of *Bgn* (encoding biglycan), *Col1a1* (procollagen‐1), *Mrc1* (encoding the macrophage‐specific mannose receptor, CD206), and the housekeeping gene *Hprt* (hypoxanthine phosphoribosyltransferase). Primer sequences were: *Bgn*, forward: 5′‐GTGGTCCAGGTGAAGTTCGT‐3′, reverse: 5′‐ACAACCGTATCCGCAAAGT‐3′; *Col1a1*, forward: 5‐GAGCGGAGAGTACTGGATCG‐3′, reverse: 5′‐GCTTCTTTTCCTTGGGGTTC‐3′; *Mrc1*, forward: 5‐ TCGCCCACCAGAGCCCACAA‐3′, reverse: 5′‐ATGCTCGCCAGCTCTCCACCT‐3′; *Hprt*, forward: 5′‐TTGCGCTCATCTTAGGCTTT‐3′, reverse: 5′‐AAGCTTGCTGGTGAAAAGGA‐3′. For quantification of *Scn5a*,* Gja1* and *Hprt* expression, we used predesigned probes: Mm00439105_m1 (*Gja1*), Mm01342518_m1 (*Scn5a*), and Mm00446968_m1 (*Hprt*). All reactions were run in accordance with the manufacturer's instructions. For both primer and probe reactions, expression of target genes was normalized to the expression of *Hprt*, run either as a primer or as a probe reaction, as detailed.

### Statistical analysis

All results are expressed as mean ± SEM. Differences between groups were statistically tested by unpaired Student's *t* test; whereas differences within groups as result of drug administration were tested using a paired Student's *t* test. Effects of isoprenaline in the two groups of mice were statistically compared using a two‐way repeated measures analysis of variance (ANOVA) with a Bonferroni post hoc *t* test when appropriate. Arrhythmia incidences were compared using a Fisher's exact test. *P* values <0.05 were considered statistically significant.

## Results

### Development of mild uremia secondary to 5/6 nephrectomy

In total, 60 mice underwent either 5/6 nephrectomy or sham operations. Of these, 45 mice survived up to 9 weeks until experiments (Fig. 1A). Body weights at the end of the study period were significantly smaller in NX mice (28 ± 1 g, *n *= 14) compared to sham‐operated mice (32 ± 1 g, *n *= 14; *P* < 0.01). Plasma contents of urea and creatinine were increased in NX mice (Fig. 1B). The concentration of calcium in the plasma was also increased in NX mice (2.8 ± 0.1 mmol/L vs. sham: 2.4 ± 0.1 mmol/L; *P* < 0.01). These data are compatible with a relatively mild uremic condition following 9 weeks of markedly reduced kidney mass, and they are comparable to earlier data from us (Madsen et al. 2017).

### No structural or mechanical remodeling of the heart in NX mice

**Figure 2:**
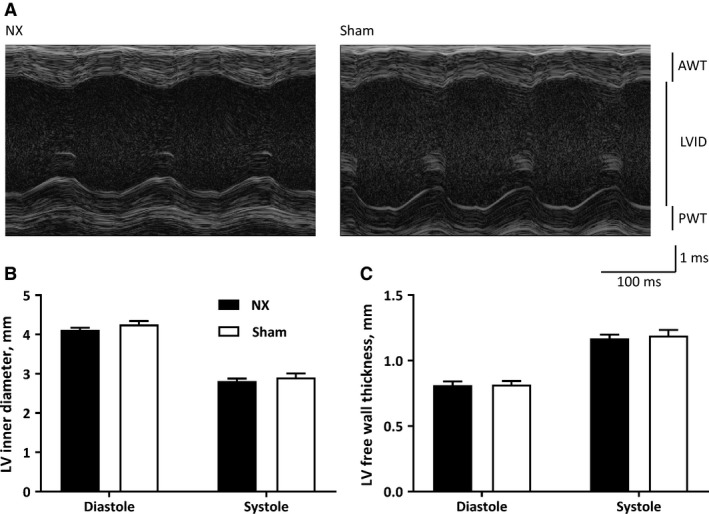
(A) Representative M‐mode echocardiographic images from the left ventricle in anesthetized mice. AWT; anterior wall thickness; LVID: left ventricular inner diameter; PWT: posterior wall thickness. (B) Left ventricular inner diameter in diastole and systole for NX and sham mice. Diastolic diameter is significantly larger than systolic diameter for both mice groups (not indicated); however, there is no difference between NX and sham. (C) Left ventricular wall thickness (mean of anterior and posterior wall) in diastole and systole for NX and sham mice. Systolic wall thickness is significantly larger than diastolic wall thickness for both mice groups (not indicated); however, there is no difference between NX and sham.

Structural and mechanical remodeling was assessed by ultrasound 8–9 weeks after nephrectomy or sham operations (Fig. 2A). Left ventricular inner diameter was comparable in both systole and diastole (Fig. 2B). In addition, left ventricular wall thickness was comparable in NX and sham mice in both systole and diastole (Fig. 2C). From these structural measurements, several mechanical indexes are estimated by the software algorithms, e.g., ejection fraction (NX: 59.8 ± 1.9 vs. sham: 60.0 ± 2.1%; *P *= 0.95) and fractional shortening (NX: 31.6 ± 1.4 vs. sham: 31.8 ± 1.4%; *P *= 0.91). During these recordings, RR intervals were comparable in the two groups of mice (NX: 478 ± 13 vs. sham: 472 ± 22; *P *= 0.81). Thus, uremia does not cause structural or mechanical cardiac remodeling in mice.

### Prolonged ventricular activation time in conscious, uremic mice after isoprenaline administration

**Figure 3:**
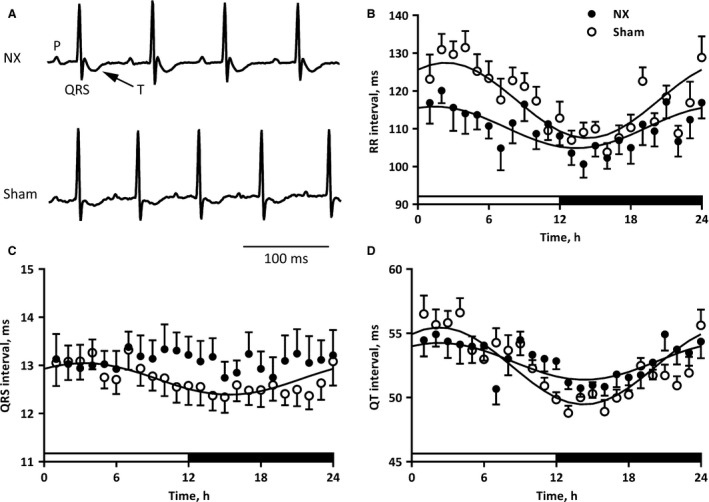
(A) Representative ECG traces from conscious NX and sham mice. *P* wave, QRS complex, and T wave are indicated on the first complex. (B) RR intervals during 24 h in NX and sham mice. The light was on from 6 am to 6 pm (0–12 h on the abscissa, indicated by a white box) and off from 6 pm to 6 am (12–24 h on the abscissa, indicated by a black box). The indicated fits are from a three‐parameter cosinor function with a fixed phase of 24 h. We tested the probability of an amplitude of zero (i.e.*,* no 24‐h rhythm), and plotted the fit only if *P* < 0.05. The amplitude of the fit, i.e., from mean to peak, is indicated on the graph. *P < 0.05 versus the amplitude of the NX fit. (C) QRS intervals during 24 h. Details as in panel B. There was no 24‐h rhythm of QRS complexes in NX mice. (D) QT intervals during 24 h. Details as in panel B.

To describe potential electrical remodeling in the uremic mice, we recorded a 1‐lead ECG for 24 h. Exemplary ECG traces are shown in Figure 3A. No arrhythmias apart from single, spontaneous premature ventricular contractions were identified in either NX or sham animals during the 24‐h recording. We did not identify any statistically significant electrophysiological differences between the 24‐h mean of the data from NX and sham mice (Table 1), although the *P* value for the statistical comparison of the RR intervals was low (110 ± 3 vs. 117 ± 2 msec in NX and sham, respectively; *P *= 0.054). The amplitude of the 24‐h rhythm in RR (Fig. 3B), PR (not shown) and QT intervals (Fig. 3D) were depressed in NX mice. Interestingly, it was not possible to fit a cosinor function to the QRS interval data from NX mice (Fig. 3C), suggesting that the natural 24‐h rhythm in QRS intervals was eliminated. The acrophase of the fit, indicating the temporal location of the peak of the electrophysiological parameter was not different in NX and sham‐operated mice.

**Table 1:** Analysis of ECG from conscious mice

	NX	Sham	*P* value
*N*, mice	10	9	–
RR interval, msec	110 ± 3	117 ± 2	0.054
PR interval, msec	37 ± 1	38 ± 1	0.39
QRS interval, msec	13.1 ± 0.4	12.7 ± 0.3	0.50
QT interval, msec	53 ± 1	52 ± 2	0.61

*P* values from a Student's *t* test.

To quantify heart‐rate variability, we identified all R waves during the 24‐h recording period. The standard deviation of the RR intervals and the standard deviation of successive RR interval differences between adjacent RR intervals were comparable in NX and sham mice. The percentage of successive RR interval differences longer than 6 msec (pRR6, (Thireau et al. 2008)) was 16 ± 2% in NX mice and 21 ± 3% in sham mice (*P *= 0.32).

**Figure 4:**
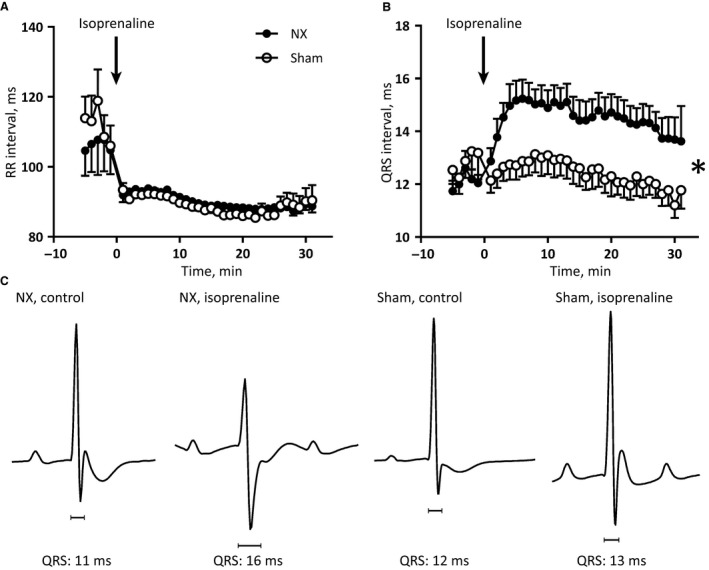
(A) RR intervals measured every minute before and after isoprenaline administration in conscious mice. (B) QRS intervals measured every minute before and after isoprenaline administration in conscious mice. * indicates a statistically significant difference between NX and sham‐operated mice based on a two‐way ANOVA on all postadministration data points. (C) Representative ECG traces from conscious NX and sham‐operated mice, before and after isoprenaline administration. Note the change in QRS morphology after isoprenaline in the NX mice, causing an overall prolongation of the QRS duration.

After the 24‐h recording, the mice received an intraperitoneal bolus injection of the *β*‐adrenergic receptor agonist, isoprenaline. Two NX mice had sustained sinoatrial‐node‐related arrhythmias with intermittent acceleration and sinus‐node block without *P* waves after isoprenaline, and were excluded from analysis. This arrhythmia incidence was not significantly different from the arrhythmia‐free sham‐operated mice (Fisher's exact test). RR intervals shortened as expected after *β*‐adrenergic receptor stimulation and this was comparable in NX and sham mice (Fig. 4A). Interestingly, QRS duration prolonged significantly in NX mice only, compared to sham mice (Fig. 4B and C). Overall, the QRS duration prolonged by 36 ± 14% in NX mice (from 12.1 ± 1.2 to 15.3 ± 1.9 msec; *P* < 0.05), but only by 7 ± 7% in sham mice (from 12.8 ± 1.9 to 13.1 ± 2.1 msec; not statistically significant). The between‐groups comparison, assessing QRS duration after isoproterenol, also showed a significantly longer QRS duration in NX mice (*P* < 0.05). To analyze this in detail and to determine the potential consequences, we performed an invasive electrophysiological study in anesthetized uremic and control mice.

### Anesthesia masks electrophysiological differences between NX and sham‐operated mice

**Figure 5:**
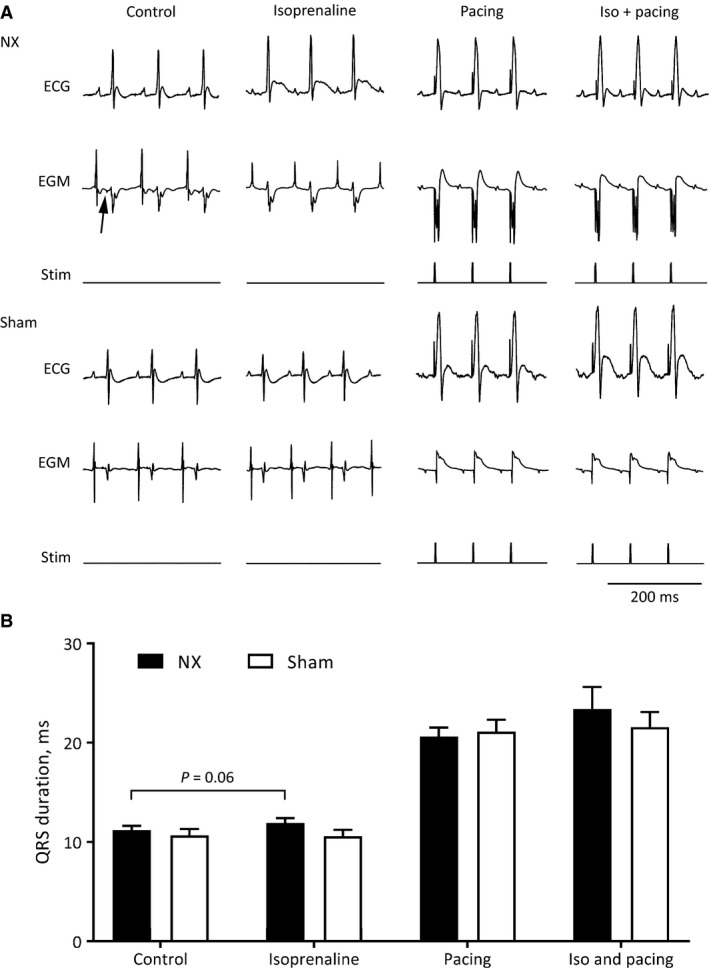
(A) Representative surface electrocardiograms (ECG) and intracardiac electrogram (EGM) from an NX and a control mice. Stimulus (stim) indicates right ventricular apex pacing. Mice were paced before and after administration of isoprenaline. Arrow points to the His potential on the EGM. (B) QRS duration in NX and control mice during normal sinus rhythm (no pacing) and during ventricular pacing, before and after administration of isoprenaline. Ventricular pacing causes a twofold prolongation of the QRS interval that was comparable in both mice groups. There is a trend toward a statistically significant isoprenaline‐induced prolongation of the QRS complex in NX mice (*P *= 0.06), but not in sham‐operated mice (*P *= 0.50).

We recorded 6‐lead surface ECGs in anesthetized NX and sham mice and the results are presented in Table 2. In the unstressed situation, we observed no differences between the two groups of mice, which confirm our findings from the conscious mice. Pacing constantly from the right ventricle at a cycle length of 80 msec produced a prolongation of the QRS complex in both NX and sham mice (to 21 ± 1 and 21 ± 1 msec, respectively; *P *= 0.75; Fig. 5). Electrical pacing to induce cardiac arrhythmia elicited short runs of ventricular tachycardia in 3/8 NX mice and 2/8 sham‐operated mice (*P *= 1.00, Fisher's exact test).

**Table 2:** Analysis of ECG from anesthetized mice

	NX	Sham	*P* value
*N*, mice	8	8	–
RR interval, msec	112 ± 5	114 ± 3	0.74
PR interval, msec	37 ± 1	39 ± 1	0.47
QRS interval, msec	10.9 ± 0.5	10.7 ± 0.4	0.78
QT interval, msec	50 ± 2	50 ± 2	0.99
J‐wave amplitude, *μ*V	125 ± 15	122 ± 18	0.87
T‐wave amplitude, *μ*V	23 ± 33	25 ± 9	0.97

Lead II was analyzed. The large SEM for T‐wave amplitude reflects that T waves presented both with positive and with negative amplitude in both NX (5 positives, 3 negatives) and sham mice (7 positives, 1 negative). J‐wave amplitude was always positive. *P* values from a Student's *t* test.

Intraperitoneal administration of isoprenaline caused a shortening in RR intervals in both NX (from 104 ± 4 to 95 ± 2 msec; *P* < 0.001) and sham‐operated mice (from 100 ± 2 to 90 ± 1 msec; *P* < 0.001, two‐way ANOVA). Simultaneously, the duration of the QRS complex changed from 11.2 ± 0.4 to 11.9 ± 0.5 (*P *= 0.06; Fig. 5B) in NX mice and from 10.7 ± 0.6 to 10.6 ± 0.6 (*P *= 0.39) in sham‐operated mice. The QRS duration was not significantly longer in NX mice compared to sham‐operated mice after isoprenaline (*P *= 0.2). Pacing to induce cardiac arrhythmias after administration of isoprenaline produced short runs of ventricular tachycardia in 5/8 NX mice and in 3/8 sham‐operated mice (*P *= 0.61). Pacing the heart 5 min after administration of isoprenaline prolonged the QRS complex in both NX and sham mice; however, to durations comparable to pacing without adrenergic stimulation (Fig. 5B). Based on these findings, we decided to test conduction velocity ex vivo.

### Ex vivo measurement of conduction velocity is comparable in NX and sham‐operated mice

**Figure 6:**
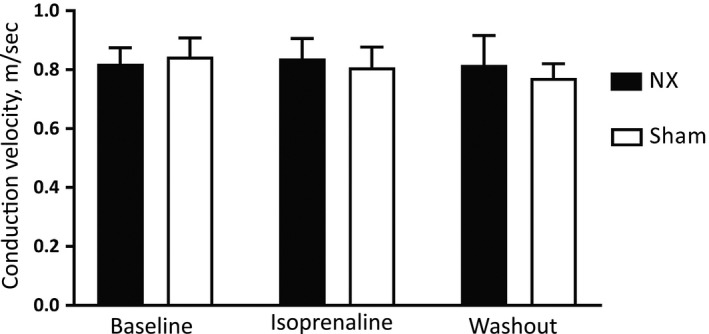
Conduction velocity in isolated right ventricular strips from NX and sham‐operated mice. Measurements were performed at baseline, under isoprenaline stimulation and after washout.

To explore the role of conduction in the observed QRS prolongation, we acutely isolated right ventricular strips from sham (*n *= 8) and NX mice (*n *= 6). On average, conduction velocity was 0.81 ± 0.07 m/sec for NX mice and 0.82 ± 0.05 m/sec for sham‐operated mice (*P *= 0.89; Fig. 6). Next, we stimulated the strips with isoprenaline (0.3 *μ*mol/L) followed by a washout phase. A two‐way ANOVA indicated no significant effect of either mouse group, treatment or their interaction.

### No difference in the expression of key genes controlling conduction velocity

**Figure 7:**
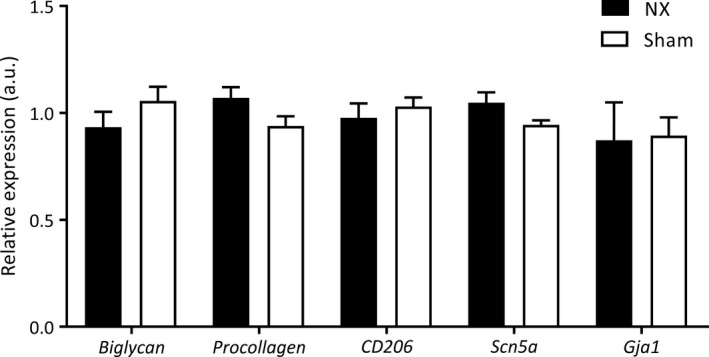
NX does not affect mRNA expression of select genes in the left ventricle of the heart. mRNA expression analyses of the fibrotic genes biglycan and procollagen‐1, the M2 macrophage marker CD206, the voltage‐gated sodium channel scn5a, and the gap‐junction protein gja1 in the left ventricle of the heart. For each gene, the relative expression (in arbitrary units, a.u.) was determined and normalized to the housekeeping gene hypoxanthine‐guanine phosphoribosyltrasferase (HPRT). One outlying data point of Gja1 expression in one NX mouse was excluded from analysis.

To determine whether the prolonged QRS complexes after uremia were corroborated by fibrotic changes in the heart, we performed gene‐expression analyses on RNA extracted from the left ventricle. mRNA expression of the profibrotic genes, *biglycan*,* procollagen‐1*, and *CD206* (a marker of profibrotic M2‐like macrophages) was similar in hearts from NX and sham‐operated mice. Likewise, mRNA expression of the cardiac voltage‐gated sodium channel (SCN5A) and the gap‐junction protein connexin 43 (GJA1) was not altered in the left ventricle from uremic as compared to sham mice (Fig. 7).

## Discussion

In summary, the present characterization of the cardiac electrophysiological consequences of CKD shows a surprisingly small cardiac impact of 5/6 nephrectomy. Structural and mechanical properties are comparable (Fig. 2) as are electrophysiological parameters at first glance (Tables 1 and 2). Only a disappearance of the 24‐h oscillation of the QRS interval duration (Fig. 3C) and an unanticipated prolongation of the QRS interval after *β*‐adrenergic receptor stimulation (Fig. 4B) in conscious NX mice are identified in the present study. There is a trend toward prolongation of the QRS complex in anesthetized NX mice after *β*‐adrenergic receptor stimulation (Fig. 5B); however, ex vivo determination of conduction velocity and analysis of expression of key genes encoding proteins implicated in conduction and fibrosis provide no underlying mechanism.

### Cardiac remodeling in animals with CKD

A rat model of CKD based on spontaneous cystic kidney disease due to an inherited samcystin protein defect has moderately elevated plasma urea levels (Hsueh et al. 2014), comparable to the levels shown in Figure 1A. (Note: The correct unit of blood urea nitrogen in Table 1 of reference (Hsueh et al. 2014) is probably mg/mL, not mg/L). In apparent conflict with our findings, Hsueh et al. (2014) find a marked ventricular hypertrophy of the hearts in rats with samcystin defect, although heart rate and cardiac pump function (e.g., ejection fraction) are comparable, as in our data. Cardiac hypertrophy has also been reported in another rat model of CKD due to partial nephrectomy (Donohoe et al. 2000a).

Comparable to our findings in mice, the samcystin‐deficient rats with CKD have unaltered refractory period and conduction velocity, and they have a normal frequency of spontaneous ventricular contractions in vivo (Hsueh et al. 2014). Notwithstanding, the hypertrophic hearts from the rats with CKD showed a statistically significant increased vulnerability to pacing‐induced arrhythmia in an isolated heart preparation (Hsueh et al. 2014), and a later study in the same rats showed that they die spontaneously after a preceding 24‐h period with a continuous decline in heart rate (Zhao et al. 2016). In the present study, we were not able to confirm increased arrhythmia susceptibility in CKD, when pacing the hearts in situ in the intact animal.

Disaggregated cardiomyocytes from rats isolated 8 weeks after 5/6 nephrectomy have larger repolarizing potassium currents and the action potentials are significantly shorter (Donohoe et al. 2000a,b), whereas cardiomyocytes from rats with 8 weeks of unilateral nephrectomy have smaller repolarizing potassium currents and longer action potentials (Lee et al. 2017). This appear to be in contrast to our present findings of unaltered QT intervals in vivo (Table 1 and 2) and to the data from Hsueh et al. (2014); however, the underlying mechanism for these differences cannot be ascertained by the data from the present study.

Fontes and colleagues report that 5/6 nephrectomy in mice cause increased plasma urea comparable to the present data. In an isolated heart preparation, they are able to induce ventricular arrhythmias in two of eight hearts, which is comparable to the data from our invasive study (five of eight mice with pacing‐induced arrhythmias; *P *= 0.3 from Fisher's exact test). Furthermore, these mice show no signs of cardiac hypertrophy. The authors further challenge the NX mice with a high‐salt diet, which increases urine albumin content; however, this does not significantly alter arrhythmia burden (Fontes et al. 2015).

Thus, it appears that isolated hearts from mice (Fontes et al. 2015) or rats (Hsueh et al. 2014) with CKD are prone to ventricular pacing‐induced arrhythmias, and the proposed mechanisms are diverse and may be influenced by model, strain, and species differences. To the best of our knowledge, the present study is the first to show that mice with CKD are not susceptible to spontaneous or pacing‐induced cardiac arrhythmias in vivo. It should be emphasized that testing the electrophysiology of the heart in vivo in the setting of CKD has the important advantage that uremic toxin exposure and potential electrolyte imbalances are intact.

### Altered QRS complex in CKD mice

We have previously documented the presence of a 24‐h rhythm of the QRS complex in conscious wild‐type mice (Gottlieb et al. 2017). The control mechanism of such 24‐h rhythm can reside in (1) the endogenous cardiac clock, using a transcription‐based mechanism (Martino and Young 2015); (2) an endogenous clock of another cell type or organ that provides a neurohumoral signal to the heart; or (3) by the central clock in the suprachiasmatic nucleus of the brain, where environmental cues entrain the master clock controlling all the peripheral organ clocks (Buhr et al. 2010). For example, disaggregated cells isolated from the heart and cultured show clear circadian rhythms in gene expression of clock genes (Durgan et al. 2005), and cardiomyocyte‐specific knockout of key clock genes leads to spontaneous heart failure (Young et al. 2014; Ingle et al. 2015). It is presently unknown what causes the 24‐h oscillation of the QRS complex; however, a rhythm in both Cx43 expression in the heart (Pizarro et al. 2013) and in SCN5A expression (Schroder et al. 2013) have been reported. It is unclear how NX could directly blunt the rhythm of expression of a gene in the heart, but it is tempting to speculate that the healthy kidney produces a signal with a 24‐h rhythm that modulates the QRS complex of the heart, and that this rhythm is lost in CKD. The signal may be a neurohumoral or it may simply be an oscillation of electrolyte concentrations in the plasma secondary to circadian rhythmicity in kidney function (Firsov et al. 2012; Sennels et al. 2012). This hypothesis would be in apparent agreement with the comparable conduction velocities we observe ex vivo (Fig. 6), where this signal would be lost or identical in both NX and sham preparations. The QRS duration increases in humans undergoing hemodialysis (Morales et al. 1998; Drighil et al. 2008; Tarif et al. 2008; Berta et al. 2012; Astan et al. 2015; Ozportakal et al. 2017), indicating that severe CKD causes an accumulation of one or more elusive factors that shorten QRS duration.

The QRS duration prolonged significantly in conscious NX mice challenged acutely with isoprenaline (Fig. 4). Moreover, in anesthetized mice, the isoprenaline‐induced increase in QRS duration was blunted and did not reach statistical significance (*P *= 0.06; Fig. 5B). Thus, it appears that isoflurane anesthesia may conceal the effect of isoprenaline on the QRS complex in NX mice, which may be related to an apparent shortening of the QRS complex under isoflurane anesthesia (comparing Tables 1 and 2). One significant caveat of comparing the two tables is that the data come from different mice and different ECG leads. Isoprenaline administration to conscious mice was performed during the light phase, where QRS duration was comparable between NX and sham‐operated mice (Fig. 3C), and the effects of isoprenaline may be different during the dark phase, where the QRS duration is shorter in the sham‐operated mice. In both anesthetized and conscious mice, the isoprenaline challenges were performed during the light phase.

Severe hypercalcemia is associated with QRS prolongation, and it is a possibility that the small increase in the concentration of calcium in the plasma of the NX mice may have contributed to the longer QRS duration in these mice. In the present study, we did not measure plasma concentrations of sodium, potassium or any humoral factors from the kidney, and it is presently unclear what underlies the increased QRS duration after isoprenaline in NX mice.

### Clinical perspectives

Ventricular arrhythmias are relatively common in patients with CKD and the reason for this is unknown (Green and Roberts 2010). The present detailed documentation of the cardiac electrophysiological effects of subtotal nephrectomy in intact, conscious mice suggests that the heart is not affected by mild uremia in mice. Notwithstanding, imbalances in neurohumoral, electrolyte or circadian systems imposed by the failing kidney may acutely change the electrophysiological parameters of the heart.

## Conclusions

Mild, chronic kidney disease does not give rise to a structural or mechanical phenotype in mice. Electrophysiologically, we identify a loss of the 24‐h diurnal rhythm in QRS duration and an unexpected prolongation of the QRS interval after *β*‐adrenergic receptor stimulation. These effects are only present in vivo, and ex vivo attempts to pinpoint the mechanism failed to do so. We conclude that normal kidney function is necessary to maintain diurnal variation in QRS duration of the heart, and that sympathetic regulation of the QRS duration is altered in kidney disease.
